# Sources of Trouble and of Joy

**Published:** 1858-07

**Authors:** 


### SOURCES OF TROUBLE AND OF JOY.

We have somewhere seen the statement, that most of our troubles arise from having a will different from that of Providence. On the other hand, Joseph John Gurney begins one of his most beautiful essays, by saying: "Much of the happiness which is here permitted to man, arises from the exercise of kindly feelings." Thus it is, that the surest way of happifying ourselves, is to practice those neighborly acts which invite out the better feelings of those around us. Exemption from trouble is a negative happiness; but to delight in removing the troubles of others, and placing smiles and gladness in their stead, is well worthy of Him who is declared to be but "a little lower than the angels." To grow old in a confiding submission to the will of God, and in the habitual exhibition of a brotherly affection to all of woman born, this is the life divine, the matchless elixir, the panacea for human sorrow, the balm of immortality.

